# Correlation of meniscus tear type with synovial inflammation and the therapeutic potential of docosapentaenoic acid

**DOI:** 10.1186/s12891-024-07491-1

**Published:** 2024-05-11

**Authors:** Lichuang Wu, Ming Ying, Yiheng Ye, Dongdong Wang, Chengwei Chen, Cailong Liu

**Affiliations:** 1 Department of Orthopaedics, The First Affiliated Hospital of Wenzhou Medical University, 1210 University Town, Wenzhou, Zhejiang 325000 China; 2 School of Pharmaceutical Sciences, Wenzhou Medical University, 1210 University Town, Wenzhou, Zhejiang 325035 China

**Keywords:** Synovitis, Meniscus tears, Inflammation, Omega-3, Docosapentaenoic acid

## Abstract

**Background:**

Synovitis, characterized by inflammation of the synovial membrane, is commonly induced by meniscus tears. However, significant differences in inflammatory responses and the key inflammatory mediators of synovium induced by different types of meniscal tears remain unclear.

**Methods:**

Magnetic resonance imaging (MRI) was employed to identify the type of meniscus tear, and the quantification of synovial inflammation was assessed through H&E staining assay. Transcription and expression levels of IL-1β and IL-6 were evaluated using bioinformatics, ELISA, RT-qPCR, and IHC of CD68 staining assays. The therapeutic potential of Docosapentaenoic Acid (DPA) was determined through network pharmacology, ELISA, and RT-qPCR assays. The safety of DPA was assessed using colony formation and EdU staining assays.

**Results:**

The results indicate that both IL-1β and IL-6 play pivotal roles in synovitis pathogenesis, with distinct expression levels across various subtypes. Among tested meniscus tears, oblique tear and bucket handle tear induced the most severe inflammation, followed by radial tear and longitudinal tear, while horizontal tear resulted in the least inflammation. Furthermore, in synovial inflammation induced by specific meniscus tears, the anterior medial tissues exhibited significantly higher local inflammation than the anterior lateral and suprapatellar regions, highlighting the clinical relevance and practical guidance of anterior medial tissues’ inflammatory levels. Additionally, we identified the essential omega-3 fatty acid DPA as a potential therapeutic agent for synovitis, demonstrating efficacy in blocking the transcription and expression of IL-1β and IL-6 with minimal side effects.

**Conclusion:**

These findings provide valuable insights into the nuanced nature of synovial inflammation induced by various meniscal tear classifications and contribute to the development of new adjunctive therapeutic agents in the management of synovitis.

**Supplementary Information:**

The online version contains supplementary material available at 10.1186/s12891-024-07491-1.

## Background

The meniscus serves a crucial function in the knee joint, encompassing weight-bearing, transmitting loads, shock absorption, joint stability, lubrication, and nutrition [1, 2]. Meniscus tear (MR), resulting from various factors such as traumatic events, degenerative processes, and sports-related activities, are among the most prevalent injuries in the knee joint [3, 4]. Additionally, MRs are significant risk factors for the occurrence and development of knee osteoarthritis (OA) [3, 5–10], a common joint disease imposing substantial psychological and economic burdens on patients and their families [11, 12]. MR can induce an inflammatory response, leading to synovitis [13], contributing to the progression of cartilage loss, and increasing the risk of knee joint pain [14]. Furthermore, evidence suggests that in patients with knee OA, synovial inflammation contributes to the disease and may play a role in the arthritis process [15–17]. Considering that MRs are classified into distinct types for precise clinical treatment [18, 19], the exploration of the association between the type of MR and the degree of synovial inflammation is important and necessary.

Synovitis, characterized by the activation of immune cells and release of inflammatory mediators, is a hallmark of several joint pathologies, including MR [13, 20, 21]. The role of synovial inflammation in pathology caused by MR has not been fully evaluated, and most studies have investigated the role of synovial inflammation in patients with high-grade synovitis and end-stage knee osteoarthritis [22, 23]. Current synovitis research is largely focused on inflammatory biomarkers in the synovium within the joint cavity [24, 25], demonstrating that, among numerous cytokines, interleukin-1 (IL-1β) and interleukin-6 (IL-6) may serve as predictive indicators of cartilage injury and synovial inflammation [26–30]. Studies in recent years have shown that the concentrations of the pro-inflammatory biomarkers IL-6 remained significantly greater in knees with chronic MRs for several months after the injury compared with normal knees, while the concentrations of IL-1β were positively correlated with those of IL-6 [31, 32]. However, changes in inflammatory biomarkers, including IL-1β and IL-6, in the synovium at different locations within the joint cavity remain inconclusive. Additionally, CD68, a glycoprotein located on the surface of macrophages, serves as a common marker for detecting the presence of these cells in tissues [33]. In synovitis, characterized by inflammation of the synovial tissue lining the joint, CD68-labeled macrophages play a crucial role in pathological detection and research [34, 35]. Their involvement in the inflammatory response and tissue damage was significant, as they released inflammatory mediators including cytokines, chemokines, and enzymes that exacerbate inflammation and contribute to tissue damage [34, 35]. Our observations revealed that synovial proliferation on the medial side was often more severe than on the lateral side for patients with medial MR. Meanwhile, cartilage damage on the injured side was often more severe than on the uninjured side. Therefore, it is reasonable to speculate that there may be differences in the expression of inflammatory factors in different areas of the synovium within the same MR. Therefore, studying the differences in the expression of inflammatory biomarkers in the synovium at different locations after MR would contribute to establishing a better understanding of the synovial microenvironment in the context of MR.

In this study, we investigate the correlation between different types of MRs and synovial inflammation, illuminating the involvement of inflammatory factor biomarkers in MR-induced synovitis. We demonstrated a positive correlation between the expression levels of IL-1β and IL-6 and the synovial inflammatory response. Additionally, we found that the degree of local inflammation is significantly higher in the anterior medial tissues than in the anterior lateral and suprapatellar regions within the same tissue. These findings enable a better understanding of the expression and distribution of inflammatory factors within the joint cavity after MR. They also allow for a more accurate prediction of the likelihood of progression to osteoarthritis after MR and potentially slowing down and preventing the progression of osteoarthritis through the inhibition of IL-1β and IL-6 expression. Furthermore, we discovered that docosapentaenoic acid (DPA), a prominent component of fish oil and widely utilized health supplement known for its abundance in various beneficial omega-3 polyunsaturated fatty acids [36–39], could mitigate synovial inflammation by inhibiting the transcription and expression of IL-1β and IL-6. These results reveal a potential biomarker for synovitis and may offer a promising drug for synovitis treatment.

## Materials and methods

### Patient population

**Fig. 1:**
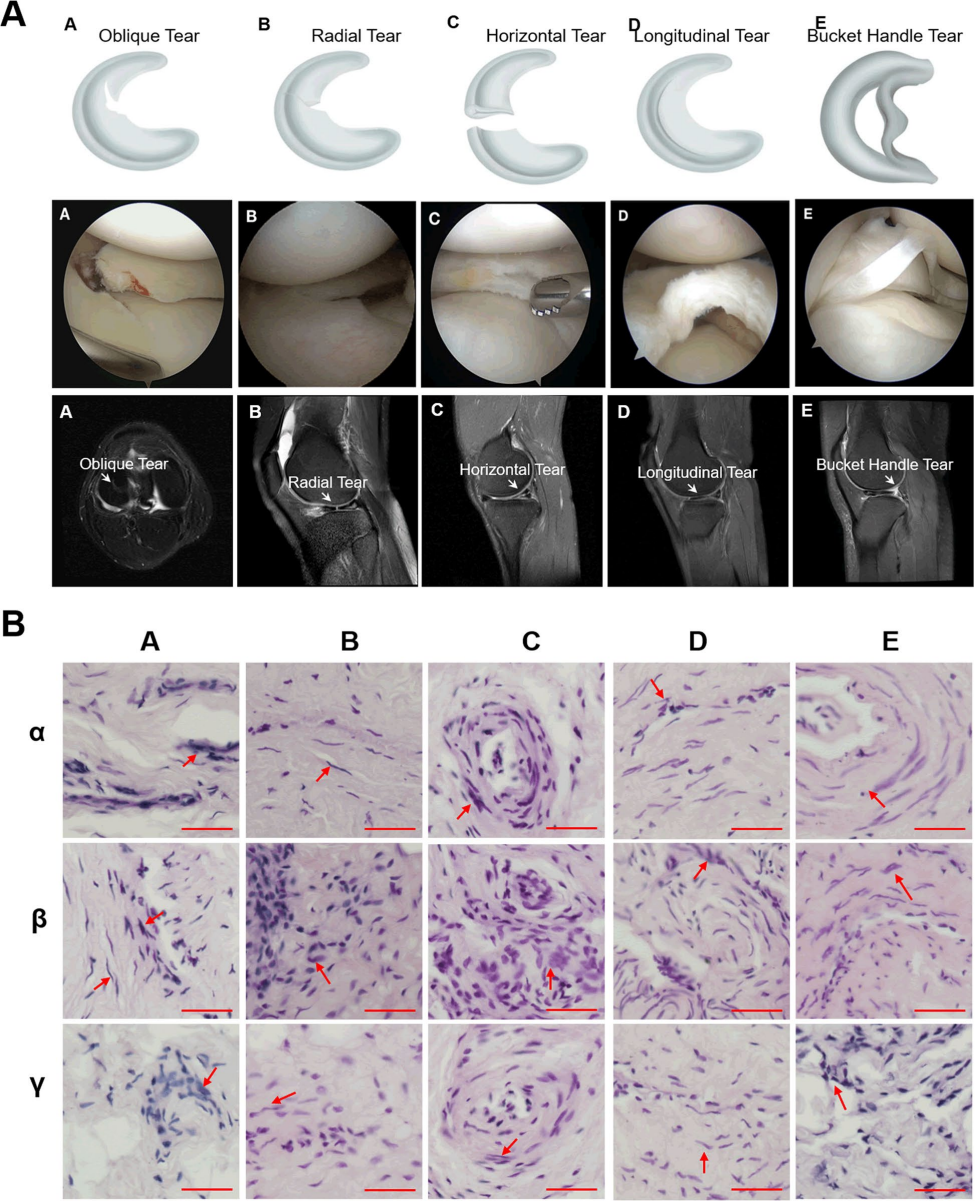
Classification of meniscal tears. **A** Meniscus tear can be divided into five types according to the different types of tear sites, namely oblique tear (**A**), radial tear (**B**), horizontal tear (**C**); longitudinal tear (**D**), and bucket handle tear (**E**). **B** Representative images of H&E staining assay of synovial inflammation induced by different meniscal injuries caused by oblique tear (**A**), radial tear (**B**), horizontal tear (**C**); longitudinal tear (**D**), and bucket handle tear (**E**), respectively. Additionally, in the same synovitis clinical sample, α represents the anterior medial region, β represents the anterior lateral region, and γ represents the suprapatellar region. Red arrow indicated cell nuclei. Scale bar, 100 µm

A total of fifteen patients (*n* = 15) with meniscus tears participated in the study. Before arthroscopy, all patients were diagnosed with meniscus tears using magnetic resonance imaging (MRI) and presented corresponding pain symptoms. The severity of meniscus tear was evaluated and confirmed through arthroscopic surgery. 15 patients were categorized into different groups based on the type of meniscus tear: oblique tear (Group A), radial tear (Group B), horizontal tear (Group C), longitudinal tear (Group D), and bucket handle tear (Group E) (Fig. 1A). Each group consisted of three patients and detailed information was presented in Table S1.

Inclusion criteria were as follows: Patients were aged 15 to 60, had a history of knee pain for 0 to 12 months, and were confirmed by MRI to have medial or lateral meniscus tear. All patients had knee osteoarthritis Kellgren-Lawrence grade 0 on X-ray, and a consensus diagnosis was obtained from two senior orthopedic surgeons.

Exclusion criteria were as follows: Patients with MRI showing both medial and lateral meniscus tears, X-ray indicating knee osteoarthritis Kellgren-Lawrence grade I-IV, those who had received immunosuppressive drugs or intra-articular sodium hyaluronate injections within the last 3 months, and patients with chronic conditions such as ankylosing spondylitis, diabetes, blood diseases, infectious diseases, rheumatoid arthritis, autoimmune diseases, malignant tumors, severe dysfunction of the heart, lungs, liver, or kidneys, or severe osteoporosis or other joint disorders.

### Synovium collection and storage

Arthroscopic surgery was conducted by experienced orthopedic surgeons for all patients. A small amount of synovial tissue was taken from the lateral and medial sides of the meniscus and the suprapatellar bursa region (the synovium sampled was the part that needed cleaning during surgery). The samples were preserved in EP tubes and stored at -80 °C. The study adhered to ethical guidelines established by the Institutional Research Human Ethical Committee (No. KY2023-R253) at The First Affiliated Hospital of Wenzhou Medical University. Patients provided written informed consent for the use of clinical biopsy specimens.

### Cells and reagents

RAW264.7 macrophage cells and human normal MIHA cells were sourced from the China Center for Type Culture Collection (Wuhan, China). RAW264.7 cells were cultured in DMEM medium (Gibco, Eggenstein, Germany) supplemented with 10% fetal calf serum (Gibco) and 100 U/mL penicillin and streptomycin (Hyclone, Logan, UT). The cells were incubated at 37 °C in a humidified atmosphere with 5% CO_2_. ELISA kits for human IL-1β and IL-6 were procured from Bioscience Inc. (San Diego, CA, USA). Lipopolysaccharide (LPS) was obtained from Sigma-Aldrich (St. Louis, MO, USA), and Docosapentaenoic acid (DPA) from Merck (#D1797).

### Bioinformatics and data analysis

The potential target genes for treating synovitis were sourced from GeneCards (https://www.genecards.org/), and the top 40 genes exhibiting the strongest correlations were identified. Transcriptome sequencing data from clinical samples of both synovitis patients and healthy individuals were retrieved from the GSE dataset in the Gene Expression Omnibus (GEO) database. R was employed for the differential analysis of transcriptome data (*p* < 0.05). Functional enrichment analysis on differentially expressed genes, encompassing KEGG pathway analysis and Gene Ontology Analysis, was conducted using the DAVID database (https://david.ncifcrf.gov/home.jsp). Statistical analyses and the visualization of raw data were carried out using Graphpad Prism and R Studio.

### ELISA determination of cytokines

The ELISA assay was conducted following a previously reported method [40]. Take 30 mg synovial clinical sample and add 4 times the volume of normal saline, then add 1% protease inhibitor PMSF (Phenylmethylsulfonyl Fluoride) and 1% protein phosphatase inhibitor. After homogenizing, remove supernatant to -80 degrees Celsius and store for later use. Aming to mitigate substrate interference, the following strategies were adopted: 1. pre-wash tissues with saline before homogenization; 2. perform tissue homogenization under chilled conditions; 3. addition of 1% PMSF and protein phosphatase inhibitors; 4. homogenize the tissues promptly and handle the samples in the shortest time. Finally, the capture antibody of IL-1β and IL-6 was diluted to 1 × using 10 × coating buffer and ddH_2_O. Subsequently, 100 μL/well of the diluted antibody was added to an ELISA plate, covered with plastic wrap, and incubated at 4 °C overnight with continuous shaking. On the following day, the plate underwent three washes with 250 μL/well 1 × PBST, followed by a final pat dry. Next, 1 × AD blocking solution (200 μL/well) was added, and the plate was incubated in a shaker at room temperature for 1.5 h. After additional washing and patting dry, IL-1β and IL-6 standard proteins with eight concentration gradients were used to construct a standard curve. The samples for measurement were introduced into designated wells and incubated at room temperature for 2 h. Post-incubation, the plate underwent washing and patting dry, followed by the addition of detection antibodies corresponding to IL-1β and IL-6, each diluted to 1 × (100 μL/well) with 1 × AD sealing solution. A one-hour incubation at room temperature in a shaker ensued. After subsequent washing and patting dry, 1 × HRP (100 μL) was added, and the plate was incubated for 30 min at room temperature. Following another round of washing and patting dry, substrate TMB (100 μL/well) was added for color development. The plate was then incubated at room temperature in the absence of light, and the reaction was promptly terminated by adding 50 μL/well of 2 M dilute sulfuric acid. The OD value was measured at 450 nm using an enzyme-labeled instrument. A standard curve was constructed, and sample concentrations were determined based on their respective OD values.

### Real-time quantitative PCR (RT-qPCR)

The RT-qPCR was conducted as previously described [41]. In summary, 50 mg of tissue or 5 × 10^7^ cells were homogenized in 800 μL Trizol. Subsequently, 200 μL of chloroform was added, followed by vigorous vortexing for 15 s. The mixture was allowed to rest on ice for 2 min at 4 degrees Celsius, and then centrifuged at 12,000 g for 15 min. The upper aqueous phase was carefully transferred to another labeled EP tube. An equal volume of isopropyl alcohol was added, thoroughly mixed, inverted 10 times, left on ice for 10 min, and centrifuged at 12,000 g for 15 min. The supernatant was discarded, and 1 mL of 75% anhydrous ethanol at ice-cold temperature was added. After gentle shaking and inversion, the solution was centrifuged at 7,500 g for 10 min. Following discarding the supernatant, the precipitate was blotted with absorbent paper, left on the paper for about 30 min, and then dissolved in 35–40 μL DEPC water. RNA concentration and purity were assessed using NANODrop. Subsequently, cDNA was synthesized by reverse transcription of the extracted RNA following the instructions in the reverse transcription kit. For RT-PCR detection, a specific amount of primers, SYBR Green, cDNA, and water were combined. Reverse transcription and quantitative PCR were performed using Power SYBR Green qPCR master mix (Invitrogen) in a LightCycler 480 (Roche) with the following specific primers (Thermo Fisher): GAPDH, 5′-GCACCGTCAAGGCTGAGAAC-3′ (forward) and 5′-TGGTGAA GACGCCAGTGGA-3′ (reverse); IL-6, 5′-ACTCACCTCTTCAGAACGAATTG-3′ (forward) and 5′-CCATCTTTGGAAGGTTCAGGTTG -3′ (reverse). IL-1β, 5′-ATGATGGCTTATTACAGTGGCAA-3′ (forward) and 5′-GTCGGAGATTCGTAGCTGGA-3′ (reverse). The housekeeping gene glyceraldyde-3-phosphate dehydrogenase (GAPDH) was used for normalization. The remaining data in different processing groups are compared with the data anterior medial region (α) for normalization processing.

### Hematoxylin and eosin staining (H&E staining assay)

The H&E staining assay was conducted following a previously reported method [42]. In brief, the acquired tissue was fixed in 4% paraformaldehyde for 48 h. Subsequently, the tissue was placed in an embedding box, washed with water for 6 h, and immersed in 70% ethanol overnight. The tissue underwent sequential soaking in 80%, 95%, and two rounds of 100% ethanol, each for specified durations. This was followed by xylene I for 15 min and xylene II for 10 min. Soft wax and hard wax were applied for 90 min each before embedding the fixed tissue into wax blocks. The paraffin-coated tissue was then sectioned into 5 μM thin slices at 42 degrees Celsius. The sections were air-dried and incubated in a 65 °C incubator. Following additional xylene treatments and a series of ethanol washes, the sections were rinsed twice with PBS for 5 min each and stored in PBS for subsequent use. Nuclei were stained with hematoxylin for 5 min, followed by two 3-min washes with distilled water. Eosin dyeing lasted for 30 s, and a 10-min distilled water rinse ensued. After dehydration, neutral resin was applied for sealing, and fluorescence microscopy was employed for observation.

### Immunohistochemical staining assay (IHC staining assay)

The IHC staining assay was conducted following a previously reported method [42]. In summary, the acquired tissue pathological sections were air-dried for 4 h, followed by dewaxing and hydration. Distilled water was used for a 3-min wash, repeated twice. The sections underwent soaking in sodium citrate buffer, high-pressure antigen retrieval for 3 min, and subsequent cooling to room temperature. A 3% hydrogen peroxide treatment at 37 °C for 10 min was applied to eliminate endogenous enzymes. Following three washes with PBS, 5% BSA was added and incubated at room temperature for 30 min to block non-specific proteins. The diluted primary antibody was applied, placed in a humidified chamber, and incubated at 4 °C overnight. After three PBS washes, a horseradish peroxidase-labeled secondary antibody was added and incubated at room temperature for 1 h. For DAB color development, the sections underwent three PBS washes, followed by incubation with the DAB color development solution, and staining intensity was observed under a microscope. Hematoxylin staining of the nuclei lasted for 1 min. After dehydration, the slides were sealed with neutral resin and examined using a fluorescence microscope. Quantitative statistics were performed by Image J software. Select RGB stack in image type, and adjust the threshold to change the selected area in image. Select required parameters in analyze. Finally, the mean value of the obtained data is analyzed.

### Cell viability assay

The cell viability was assessed by the 3-(4,5-dimethythiazol)-2,5-diphenyltetrazolium bromide (MTT) assay as described previously [41]. Briefly, RAW264.7 macrophages (3000 cells/well in 96-well plates) were incubated at 37 °C overnight. The compound DPA was dissolved in DMSO and then diluted in medium to the desired final concentration. Cells were treated with or without DPA for 48 h and then added MTT (0.5 mg/mL) 20 μL/well followed by another 4 h. The reaction product formazan was dissolved in 100 μL DMSO after discarding the culture medium. The cell viability was determined by reading the absorbance at 490 nm by the spectrophotometer (DTX880, Beckman Coulter, CA, USA). Results are presented as the mean of three measurements ± standard deviation (*n* = 3).

### Colony formation assay

The colony formation assay was conducted as previously described [41]. Human normal MIHA cells were plated in 12-well dishes at a density of 600 cells per well and incubated overnight for cell adhesion. Subsequently, the cells were cultured in standard growth media for 10 days, during which they were exposed to varying concentrations of docosapentaenoic acid (DPA) or 0.01% DMSO (Control) to assess colony growth. Following a triple rinse with phosphate-buffered saline (PBS), the colonies were fixed with 4% formaldehyde for 15 min and then stained with 0.04% crystal violet for an additional 15 min. After two washes with distilled water, the colonies were examined using a light microscope. Colony counts were conducted in three independent experiments.

### EdU (5-ethynyl-2′-deoxyuridine) staining assay

The proliferation of human normal MIHA cells was evaluated using the EdU staining proliferation kit (Beyotime, China). Cells were plated on coverslips in 12-well plates at a density of 3 × 10^4^ cells per well. They were then treated with different concentrations of docosapentaenoic acid (DPA) or 0.01% DMSO (Control) for 48 h and subsequently observed using a Nikon fluorescence microscope.

### Statistical analysis

Statistical analyses were performed with GraphPad (GraphPad Prism) using a two-tailed Student *t* test or one-way or two-way analysis of variance (ANOVA). Values are presented as mean ± standard deviation (SD)(*, *p* < 0.05; **, *p* < 0.01; ***, *p* < 0.001). *p* values were calculated using GraphPad. *p* < 0.05 was considered to be statistically significant.

## Results

### Diverse synovial pathological morphologies induced by varied meniscal tears

**Fig. 2:**
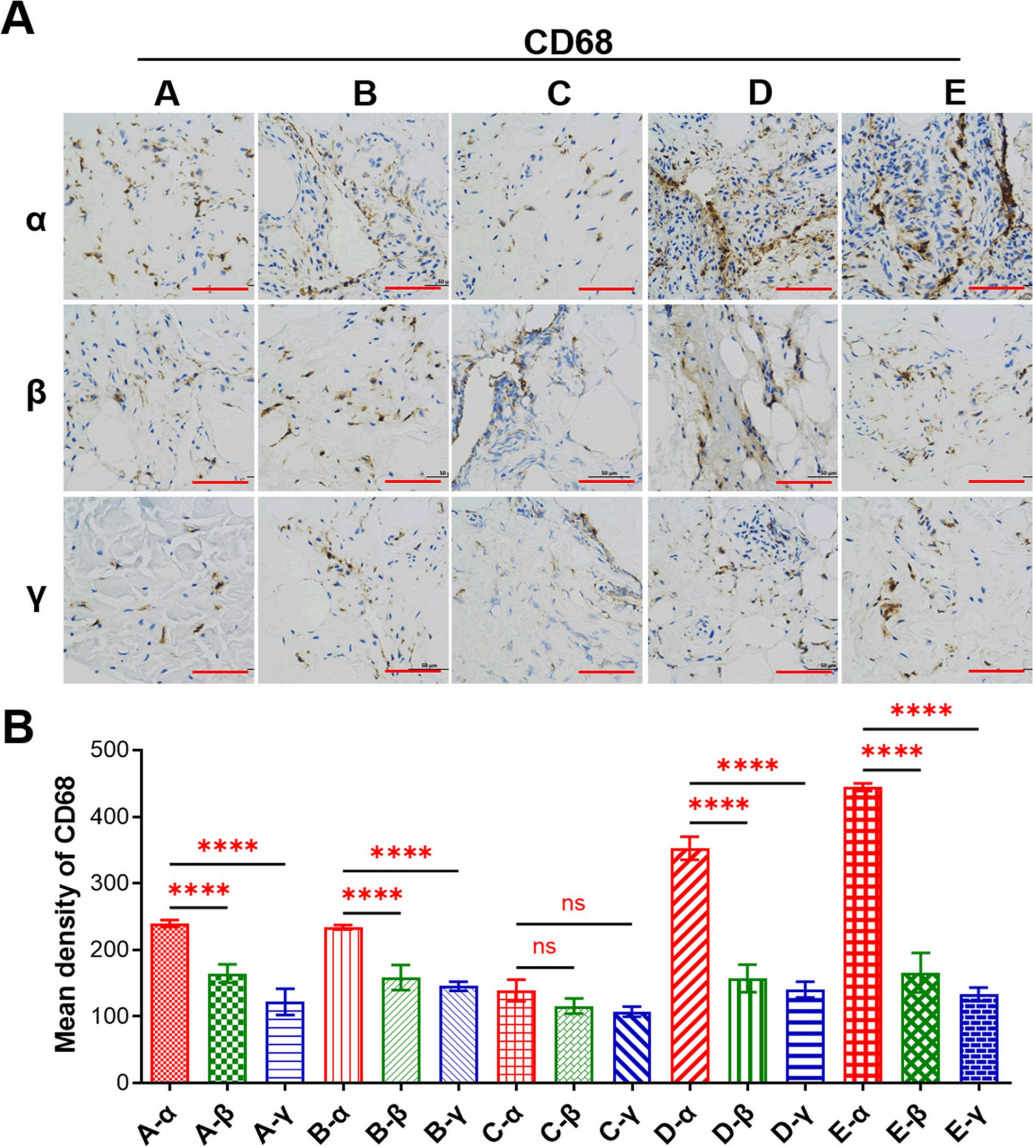
Degree of macrophage infiltration in clinical synovitis tissue. **A** Representative images of IHC of CD68 in clinical synovitis tissue. **A** refers to synovitis are caused by oblique tear in the meniscus, **B** refers to radial tear, **C** refers to horizontal tear, **D** refers to longitudinal tear, and E refers to bucket handle tear. Additionally, in the same synovitis clinical sample, α represents the anterior medial region, β represents the anterior lateral region, and γ represents the suprapatellar region. **B** Quantification of data in **A**. Values are the average ± SD of three independent experiments. p values were calculated using the two-way analysis of variance (ANOVA) (ns, not significant, ****p < 0.001). Scale bar, 100 µm

From the 15 patients enrolled in the study between March 2022 and March 2023, comprising 9 males and 6 females participated in the study. The average age was 36.5 (range, 15 to 58), with 7 cases in the right knee, 8 cases in the left knee, 11 cases of lateral meniscus injuries, and 4 cases of medial meniscus injuries. The average time since injury to surgery was 7.47 months (range, 0.33 to 12 months). The preoperative average VAS score was 2.87 (range, 2 to 4). We chose Lysholm score [43] to evaluate joint function, which averaged 71.4 (range, 62 to 79). The causes of knee injury and history of knee swelling were recorded. Detailed information is presented in Table S1. Subsequently, the pathological differences among various types of meniscus tears were assessed using the H&E staining assay (Fig. 1B). The degree of synovial inflammation was carried out on haematoxylin and eosin (H&E)-stained slides.The values of the parameters were summarized and interpreted as follows: 0–1, no synovitis; 2–4, low-grade synovitis; and 5–9, high-grade synovitis [22]. Five patients got score 1, six patients got score 2, and four patients got score 3 from the enrolled patients. Detailed information is presented in Table S1. The results revealed that synovial tissues in cases of Group C exhibited no significant lesions, featuring tightly arranged cell nuclei, intact cellular structures, clear nuclear membranes, and visible chromatin (Fig. 1B). Conversely, synovial tissues in cases of Groups B and D showed some damage, with cell nuclei exhibiting lighter staining and some undergoing nuclear division (Fig. 1B). In contrast, synovial tissues in cases of Group A and E displayed significant abnormalities, including unclear nuclear membranes and chromatin boundaries, deformation, and loss of normal cellular structure, indicating severe damage (Fig. 1B). Additionally, the H&E staining assay demonstrated that the impact of the same meniscus tear on different regions of the synovial tissue was more pronounced in the anterior medial region compared to the suprapatellar and anterior lateral regions (Fig. 1B). To assess the extent of inflammation, the degree of macrophages infiltration in synovitis tissues were analyzed and quantified. The results showed that the brown-yellow points, which indicated CD68 positivity, in Group C were less, while the brown-yellow points in Group A, B and D were increased to varying degrees, suggesting an increase in macrophage infiltration in these groups. Among them, Group E had the highest amount of infiltration (Fig. 2A). Quantitative statistical results also confirmed this conclusion (Fig. 2B).

### IL-1β and IL-6 maybe promising markers of meniscal injury-induced synovial inflammation

**Fig. 3:**
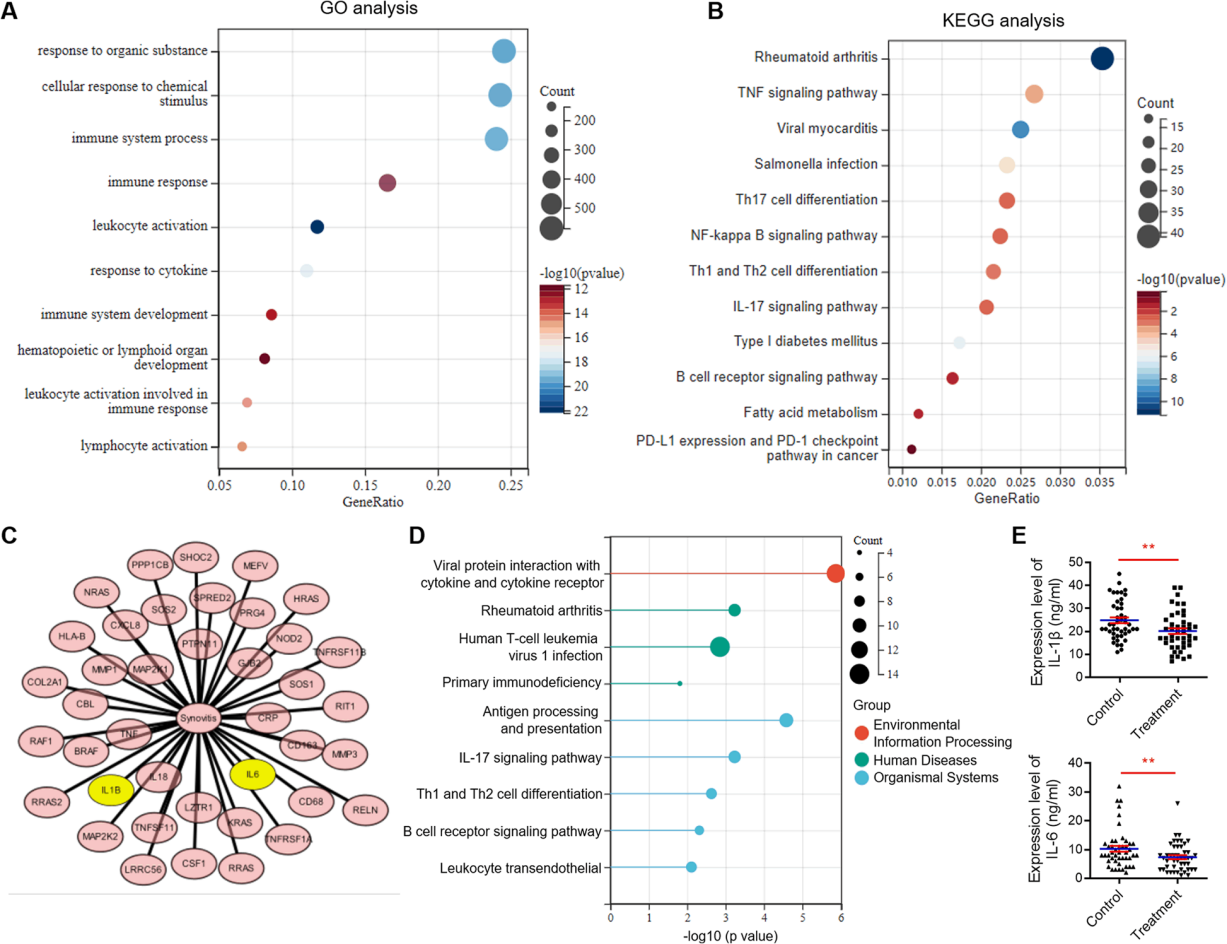
GO and KEGG analyses of GSE46750 and GSE198520 dataset. **A** Top 10 enriched GO terms for biological process of GSE46750 dataset. The color of each bubble represents the *p*-value, while the bubble size represents the gene number. **B** Top 10 enriched KEGG terms of GSE46750 dataset. The color of each bubble represents the *p*-value, while the bubble size represents the gene number. **C** Significant variations in the expression levels of IL-1β and IL-6 were observed before and after the clinical treatment among 42 genes exhibiting the most robust correlation with synovitis treatment targets according to the GSE46750 dataset. **D** Top 10 enriched GO terms of GSE198520 dataset. The color of each bubble represents the *p*-value, while the bubble size represents the gene number. **E** The expression of IL-1β and IL-6 in synovial tissue before and after treatment were statistically significant in GSE198520 dataset. Values are the average ± SD of three independent experiments. *p* values were calculated using the unpaired student’s *t*-test (**p < 0.01)

A bioinformatics analysis, utilizing the GSE46750 dataset, was conducted to identify potential markers of synovial inflammation induced by meniscal tear. The top 10 significantly enriched terms derived from GO and KEGG analyses revealed pronounced overexpression and activation of inflammatory factors and their associated signaling pathways in synovial cells from inflammatory areas of osteoarthritis synovial membrane (Fig. 3A, B and C). These findings indicated that both IL-1β and IL-6 hold promise as diagnostic and therapeutic markers for synovitis. This assertion was further supported by bioinformatics analysis of the GSE198520 dataset, which demonstrated a notable reduction in overall synovial inflammation levels, particularly in the expression of IL-1β and IL-6, following treatment (Fig. 3D and E). In conclusion, these results suggested that IL-1β and IL-6 might serve as promising markers for meniscal tear-induced synovial inflammation.

### IL-1β and IL-6 expression correlated with the region of synovitis and the formation inducement

**Fig. 4:**
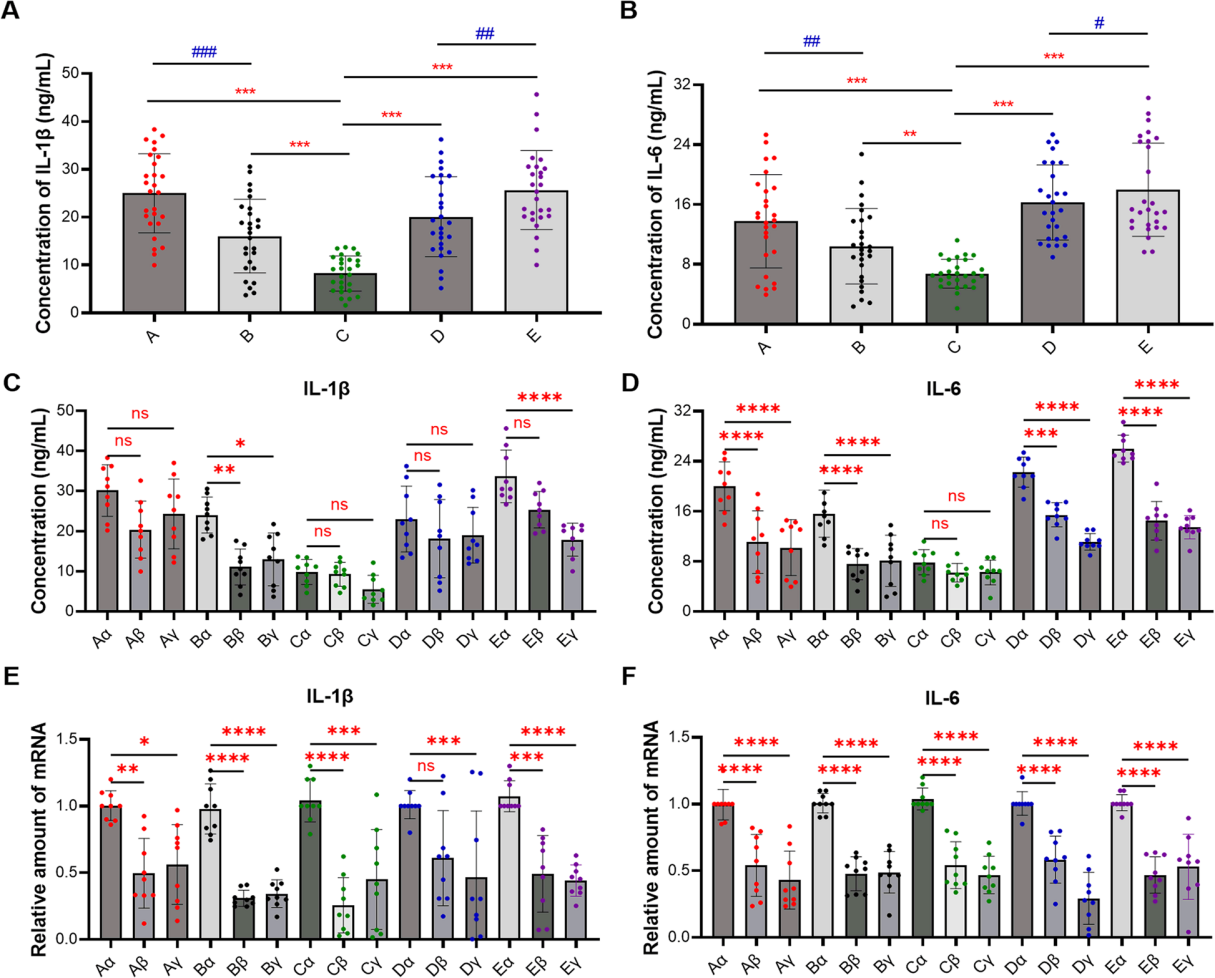
Transcription and expression levels of IL-1β and IL-6 in clinical synovitis tissue. **A** and **B** ELISA determined the abundance of IL-1β protein (**A**) and IL-6 protein (**B**) in clinical synovitis tissues caused by different types of meniscal tears. **C** and **D** ELISA determined the protein abundance of IL-1β (**C**) and IL-6 (**D**) in different region. **E** and **F** RT-qPCR determined the transcription levels of IL-1β and IL-6 in clinical samples. **A** refers to synovitis is caused by oblique tear in the meniscus, **B** refers to radial tear, **C** refers to horizontal tear; **D** refers to longitudinal tear, and **E** refers to bucket handle tear. Additionally, in the same synovitis clinical sample, α represents the anterior medial region, β represents the anterior lateral region, and γ represents the suprapatellar region. Values are the average ± SD of three independent experiments. *p* values were calculated using the two-way analysis of variance (ANOVA) (ns, not significant, *p < 0.05, **p < 0.01, ***p < 0.001, ****p < 0.0001 or ^#^*p* < 0.05, ^##^*p* < 0.01, ^###^*p* < 0.001)

The subsequent experiments examined the transcription and expression of IL-1β and IL-6 in the aforementioned clinical synovitis tissue samples using ELISA, IHC, and RT-qPCR assays. ELISA results indicated that among the tested groups, the concentration of the inflammatory factor IL-1β was highest in Group A and E, followed by Group B and D, with the lowest concentration observed in Group C (Fig. 4A). Concurrently, the concentration of IL-6 exhibited a similar distribution trend among these groups (Fig. 4B). Collectively, these results demonstrated a significant expression difference of IL-1β and IL-6 between Groups A, B, D, E and Group C. Moreover, the expression levels of IL-1β and IL-6 in Groups AE were significantly higher than those in Groups BC. Additionally, our results indicated that the expression of IL-1β and IL-6 varied among different synovial tissue sections within the same type of synovitis, and its expression in the anterior medial region was significantly higher (Fig. 4C and D). Moreover, RT-qPCR results suggested that in all types of synovitis caused by meniscal tears, the transcription level of IL-1β in the anterior medial region was higher than that in the anterior lateral and suprapatellar regions, and this difference was statistically significant (Fig. 4E). A similar trend was observed in the transcription levels of IL-6 (Fig. 4F).

**Fig. 5:**
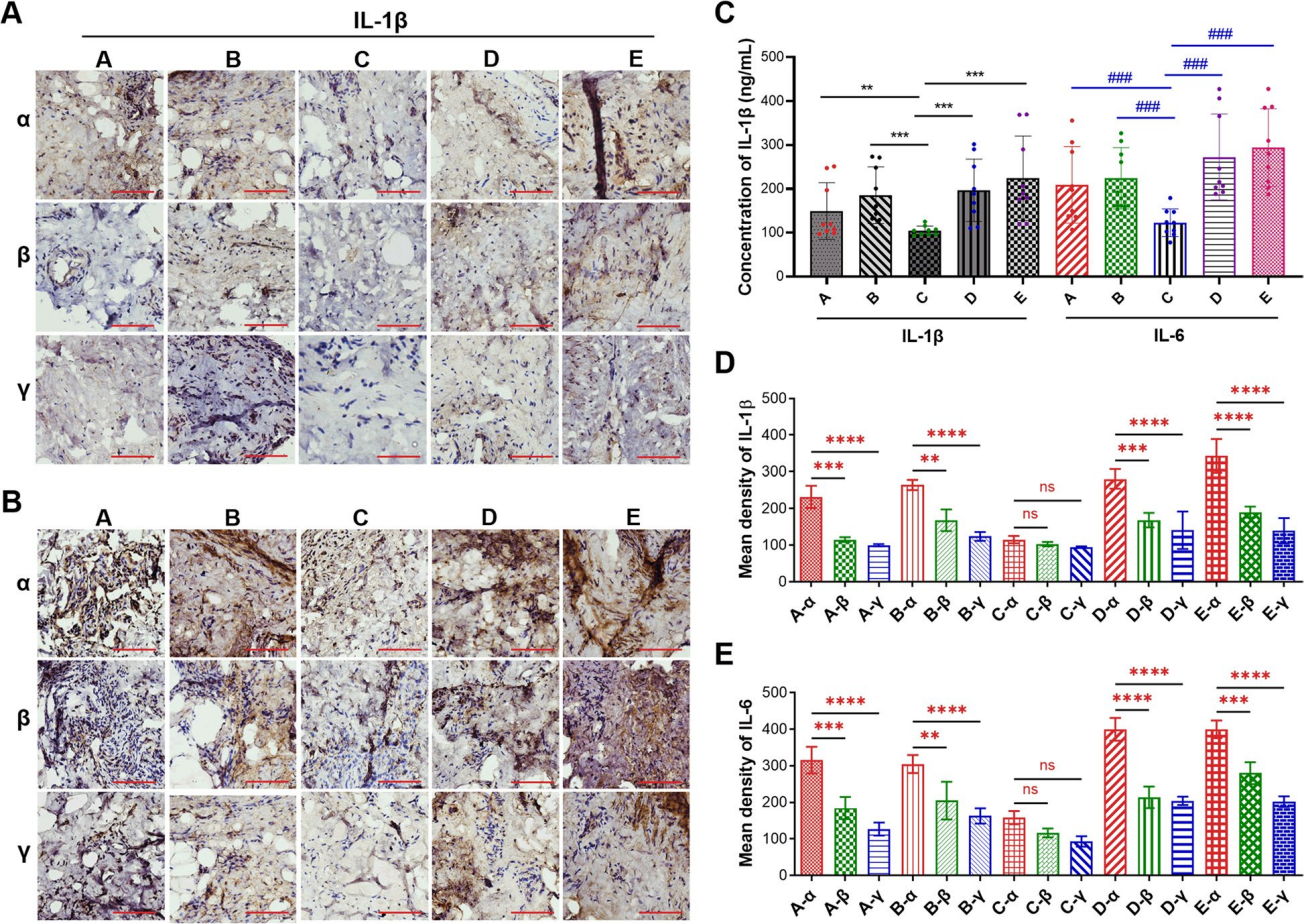
The abundance of IL-1β and IL-6 in clinical synovitis tissue. **A** and **B** Representative images of IHC of IL-1β (**A**) and IL-6 (**B**) in clinical synovitis tissue. **C-E** Quantification of data in **A** and **B** respectively. Values are the average ± SD of three independent experiments. *p* values were calculated using the two-way analysis of variance (ANOVA) (ns, not significant, **p < 0.01, ***p < 0.001, ****p < 0.0001 or ^###^*p* < 0.001). Scale bar, 100 µm

To further investigate these synovial tissue inflammation levels, immunohistochemistry (IHC) experiments were conducted using IL-1β and IL-6 antibodies. Representative image of IHC, the results revealed fewer positive points in Group C, while Groups A, B and D exhibited varying degrees of increased positivity in clinical synovitis tissue and corresponding areas, suggesting an augmentation in the infiltration of inflammatory factors in these groups. Particularly, Group E displayed the highest positivity rate, indicating the most prominent inflammation expression (Fig. 5A and B). Further quantitative statistical results confirmed this conclusion (Fig. 5C). Consistent with the ELISA data, the quantitative analysis of Fig. 5A and B showed that in the clinical tissues of the same synovitis, the expression level of the inflammatory factor IL-1β and IL-6 in the anterior medial region was significantly higher than in the anterior lateral and suprapatellar regions (Fig. 5D and E). These findings collectively supported the notion that IL-1β and IL-6 expression correlated with the region of synovitis and the formation inducement.

### IL-1β and IL-6 expression in the anterior medial tissues had the most important clinical significance

**Fig. 6:**
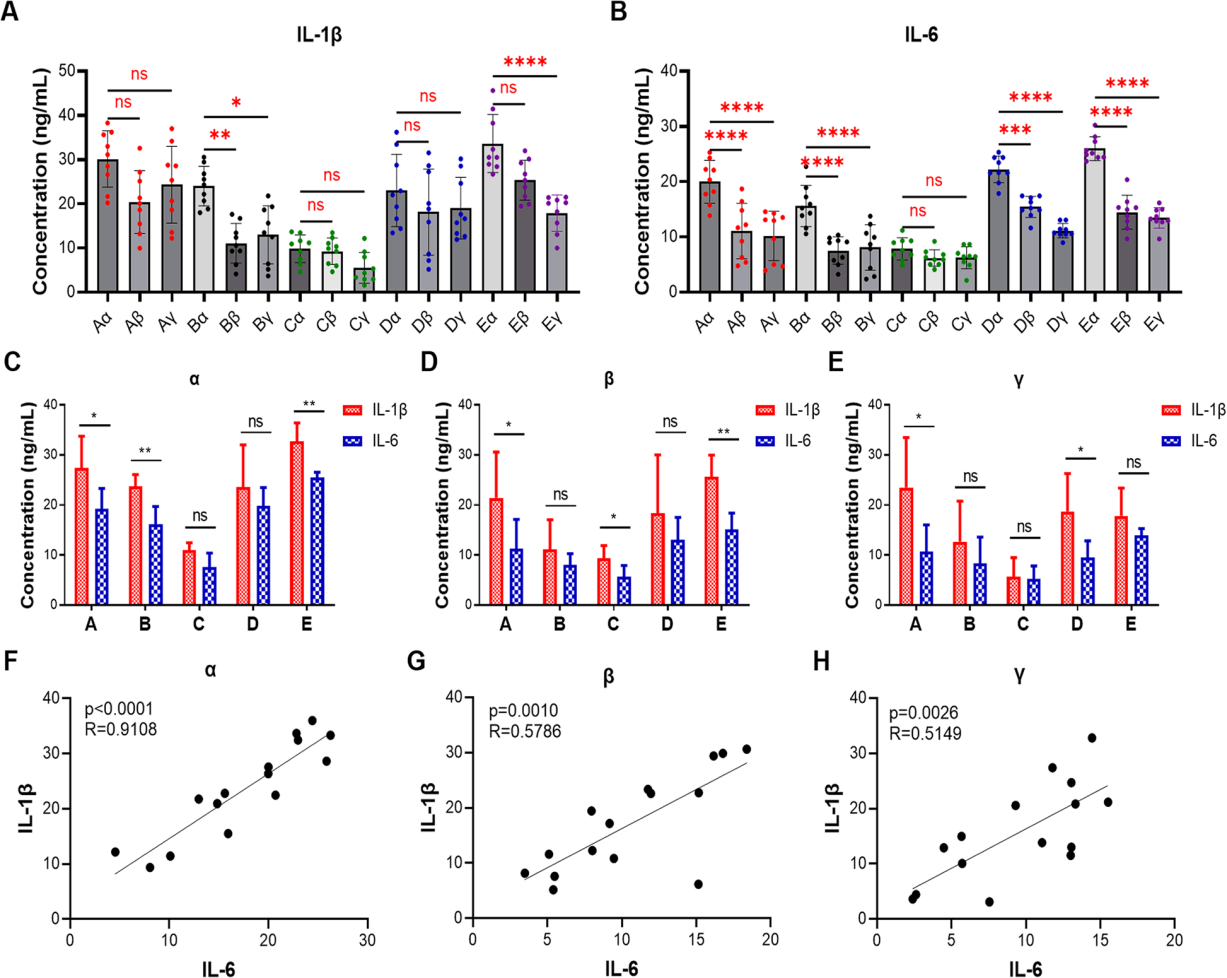
The correlation between IL-1β and IL-6 in different region of clinical synovitis tissue. **A** and **B** ELISA determined the expression levels of IL-1β (**A**) and IL-6 (**B**) in different region of clinical synovitis tissue causing by different meniscus tears. **C** The abundance of IL-1β and IL-6 in the anterior medial region of synovitis induced by different meniscal tears were determined by ELISA assay. **D** The abundance of IL-1β and IL-6 in the anterior lateral region of synovitis induced by different meniscal tears were determined by ELISA assay. **E** The abundance of IL-1β and IL-6 in the suprapatellar region of synovitis induced by different meniscus tears were determined by ELISA assay. **F** The correlation of IL-1β and IL-6 in the anterior medial region of synovitis. **G** The correlation of IL-1β and IL-6 in the anterior lateral region of synovitis. **H** The correlation of IL-1β and IL-6 in the suprapatellar region of synovitis. Values are the average ± SD of three independent experiments. *p* values were calculated using the unpaired student’s *t*-test and one-way analysis of variance (ANOVA) (ns, not significant, *p < 0.05, **p < 0.01, ***p < 0.001, ****p < 0.0001). The R values in F, G and H were calculated using the Pearson correlation analysis

The above results prompted us to examine the clinical significance of IL-1β and IL-6 expression in the anterior medial tissues. ELISA data indicated that in synovitis caused by different types of meniscus tears, the expression levels of IL-1β and IL-6 in the anterior medial region were not only the highest compared to the anterior lateral and suprapatellar regions but also exhibited higher consistency than in the anterior lateral and suprapatellar regions (Fig. 6A and B). Subsequently, the Pearson correlation analysis also indicated that IL-1β and IL-6 exhibited the highest correlation (*R* = 0.9108) in the anterior medial region compared to the anterior lateral and suprapatellar regions (Fig. 6C-H). Taken together, these findings confirmed that IL-1β and IL-6 expression in the anterior medial tissues holds paramount clinical significance.

### Docosapentaenoic acid (DPA) inhibited the transcription and expression of IL-1β and IL-6 with limited side-effect

**Fig. 7:**
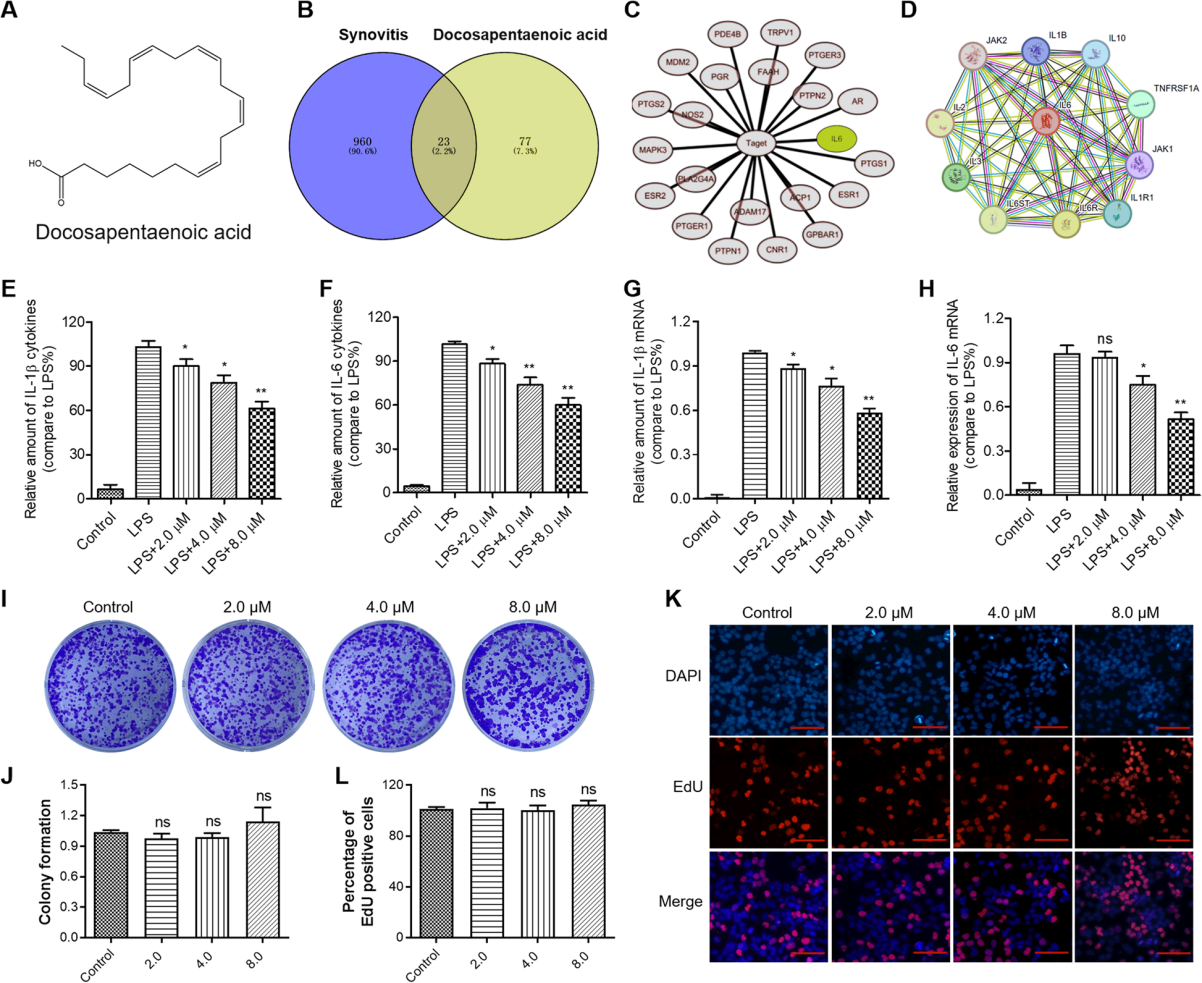
Docosapentaenoic acid (DPA) inhibited the transcription and expression of IL-1β and IL-6 without any side-effect. **A** The molecular structure of DPA. **B** Venn diagram depicting overlaps of common targets between synovitis and DPA. **C** The drug–common targets interconnection network of DPA against synovitis. **D** Protein–protein interaction network of IL-6. **E** and **F** DPA decreased the abundance of IL-1β and IL-6 protein in a dose-dependent manner. **G** and **H** DPA inhibited the transcription of IL-1β and IL-6 in a dose-dependent manner. **I** DPA had no inhibitory effect on the colony formation of human normal liver MIHA cells. **J** Quantitative analysis of the results shown in **I**. **K** DPA had no inhibitory effect on the DNA synthesis of human normal liver MIHA cells. **L** Quantitative analysis of the results shown in **K**. Values are the average ± SD of three independent experiments. *p* values were calculated using the one-way analysis of variance (ANOVA) (ns means no significant with *p* > 0.05, *p < 0.05, **p < 0.01). Scale bar, 100 µm

To assess the potential therapeutic impact of DPA (Fig. 7A) on synovitis, we conducted network pharmacology analysis. We obtained information on known synovitis treatment targets from DisGeNET and potential DPA targets from PharmMapper. A Venn diagram revealed 23 common targets, as shown in Fig. 7B. Particularly, IL-6 was among these common targets, as illustrated in Fig. 7C. Using a protein network interaction map, we observed that IL-6 was closely linked to several inflammatory factors, including IL1B, IL2, IL3, IL10, as depicted in Fig. 7D. Consequently, we speculated that DPA, as an active component in fish oil, might serve as a potential agent for synovitis treatment. Experimental data demonstrated that DPA treatment effectively alleviated the transcription and expression levels of IL-1β and IL-6 induced by LPS stimulation in RAW264.7 macrophages (Fig. 7E-H). Furthermore, data obtained from ELISA and RT-qPCR assays suggested that the inhibitory effect of DPA was dose-dependent, As the half inhibitory concentration (IC50) of DPA against RAW264.7 macrophages viability was 18.46 ± 2.12 µM, the concentrations 2, 4, 8 µM of DPA was chose in the following experiments (Fig. 7E-H). Under the consideration that hepatotoxicity and hepatic injury were important safety issues considered in drug development in the pharmaceutical industry [44–46], and normal human liver cells (MIHA) were the most commonly used liver cell [47–49]. The safety profile of DPA was further assessed using colony formation and EdU staining assays on MIHA cells. Results indicated that the number of normal MIHA cell colonies in the DPA (2.0, 4.0, or 8.0 µM)-treated group was comparable to that in the control group (Fig. 7I), and no significant difference was observed between them (Fig. 7J). Moreover, data from the EdU assay further verified the impact of DPA on MIHA cell proliferation. As shown in Fig. 7K and L, DPA treatment did not decrease the number of EdU-positive cells These results indicated that DPA had almost no side-effect on normal cells.

## Discussion

Meniscus tears have been identified as an orthopedic epidemic, resulting in significant adverse effects on both patient health and society [3, 18]. Considering the crucial function of the meniscus, beyond early meniscectomy, new therapies have been developed to relieve pain and prevent early degeneration of the knee joint [50–52]. However, these existing treatments still fail to meet clinical needs, which may be attributed to the complexity of meniscal tears [53]. Since meniscal tear-induced synovial inflammation is the main cause of pain and can further accelerate degeneration [54–56], investigating the association between different meniscal tears and synovial inflammation is urgent and holds important clinical value. In this study, our findings suggest that different types of meniscal tears result in variations in synovial inflammation. Moreover, our results suggest that IL-1β and IL-6 are pivotal inflammatory factors in meniscal tears-induced synovitis. To some extent, these findings are crucial for better understanding the underlying mechanisms of each meniscal tear type and guiding a prioritized treatment strategy.

The local mechanical environment of the meniscus and articular cartilage surface is altered after meniscus tear, partial tear of the medial or lateral meniscus can lead to significant increase in intra-articular contact pressure, meanwhile the medial meniscus transmits between 40 and 80% of the load across the knee [57]. Therefore, we speculate that the increased stress on the medial region of the joint is higher than that on the lateral region after meniscus tear, which may also be one of the reasons for the higher inflammation in the anterior medial region as shown in our study.

The results also indicate that, compared to the anterior lateral and suprapatellar regions, the anterior medial tissues exhibit the most severe inflammatory response and macrophage infiltration under the same meniscus tear. The identification of varying inflammatory levels in different anatomical regions of synovial tissues highlights the importance of considering regional variations in both the diagnosis and treatment of synovitis. Meanwhile, elucidating the differential impact on various regions of meniscal tears-induced synovitis would contribute to more precise clinical assessments. Furthermore, the significantly higher inflammation in the anterior medial tissues along with the highest correlation (*R* = 0.9108) between IL-1β and IL-6 implies potential clinical relevance and suggests a need for targeted evaluation and management in this specific region. Understanding such regional disparities could aid in refining diagnostic and therapeutic approaches.

Omega-3 fatty acids, important nutrients, play crucial roles in obesity, neural function, cancer, and inflammation [36, 58, 59]. We discovered that DPA, a main component of omega-3 fatty acids, had a potential therapeutic effect on synovitis by significantly inhibiting the transcription and expression of IL-1β and IL-6 in RAW264.7 macrophages. Additionally, our results showed that the effective dose of DPA had almost no inhibitory effect on the cell proliferation and DNA replication of human normal liver MIHA cells, demonstrating that it had almost no side effect on human normal liver MIHA cells. Taken together, the effective inhibition ability of IL-1β and IL-6, and the absence of obvious side effects further support DPA as a promising agent for synovitis treatment.

One major limitation of this study was the limited number of clinical samples for each meniscus tear, along with the small sample size. Due to the small sample size, another limitation of this study was that there was no sufficient evidence to indicate whether there was a sex-difference. Thus, our further research will focus on the longitudinal assessments of our findings with a larger clinical sample, as well as the biosecurity and bioavailability of DPA.

## Conclusions

In conclusion, our study offers comprehensive insights into the association between synovial inflammation and different classifications of meniscus tears. We identified IL-1β and IL-6 as the key inflammatory markers, with the anterior medial tissues exhibiting the most severe inflammatory response among synovial tissues. Furthermore, we uncovered the potential therapeutic effects of DPA.

### Supplementary Information

Supplementary Material 1.

## Data Availability

Data is provided within the manuscript and supplementary information files.

## Abbreviations

DPA: Docosapentaenoic acid
OA: Osteoarthritis
IL-1β: Interleukin-1
IL-6: Interleukin-6
MR: Meniscus tear
MRI: Magnetic resonance imaging
RT-qPCR: Real-time quantitative PCR
H&E: Hematoxylin and eosin staining
IHC: Immunohistochemical
IC_50_: Half inhibitory concentration
PBS: Phosphate-buffered saline
EdU: 5-Ethynyl-2′-deoxyuridine
SD: Standard deviation
