# Supplementary material for: Correlation of meniscus tear type with synovial inflammation and the therapeutic potential of docosapentaenoic acid
Source: BMC Musculoskelet Disord. 2024 May 11;25:375. doi: 10.1186/s12891-024-07491-1 (PMC11088038; doi:10.1186/s12891-024-07491-1)
Supplement: Supplementary file 1 — Supplementary Material 1. [file 12891_2024_7491_MOESM1_ESM.docx]

**Correlation of Meniscus Tear Type with Synovial Inflammation and the** **Therapeutic Potential of Docosapentaenoic Acid**

Lichuang Wu^1,†^, Ming Ying^2,†^, Yiheng Ye^1^, Dongdong Wang^1^, Chengwei Chen^1^, Cailong Liu^1,^*

^1^ Department of Orthopaedics, The First Affiliated Hospital of Wenzhou Medical University, 1210 University Town, Wenzhou, Zhejiang 325000, China.

^2^ School of Pharmaceutical Sciences, Wenzhou Medical University, 1210 University Town, Wenzhou, Zhejiang 325035, China.

^†^Both authors contribute equally to this work.

* Corresponding author:

Cailong Liu, Ph.D, Professor

Department of Orthopaedics, The First Affiliated Hospital of Wenzhou Medical University, 1210 University Town, Wenzhou, Zhejiang 325035, China.

Tel: (+86)-577- 55578033; Fax: (+86)-577- 55578033

1. mail: liucailong@wzhospital.cn

**Table S1.**  **Patient's information and data.**

In the column of "Classification of meniscal tears"，A: oblique tear in the meniscus, B: radial tear, C: horizontal tear; D : longitudinal tear, E: bucket handle tear. Severity evaluation of meniscus tear was graded on a scale of 0 to 4, with 0=normal, 1=longitudinal surface striations, 2=longitudinal surface tears, 3=penetrating longitudinal tears producing a loose piece of tissue attached at both ends (a bucket handle tear), and 4=transverse tears producing a loose tissue flap, fibrillation of the entire meniscus, or absence of the meniscus. The degree of synovial inflammation was carried out on haematoxylin and eosin (H&E)-stained slides.The values of the parameters were summarized and interpreted as follows: 0-1, no synovitis; 2-4, low-grade synovitis; and 5-9, high-grade synovitis.

| Patient number | Injured Knee | Injured Meniscus | Classification of  meniscal tears | Sex | Age（years） | Severity evaluation results of meniscus tear | Cause of  knee injury | VAS  Score | Lysholm  Score | Length of time between injury and surgery  （months） | History of knee swelling  (months) | the degree of synovial inflammation |
| --- | --- | --- | --- | --- | --- | --- | --- | --- | --- | --- | --- | --- |
| 1 | Right | Lateral | A | Female | 34 | 4 | Sprained while walking | 2 | 72 | 1 | 1 | 2 |
| 2 | Left | Lateral | A | Male | 15 | 4 | Sprained while playing basketball | 3 | 65 | 1 | 1 | 2 |
| 3 | Right | Lateral | A | Male | 23 | 4 | Fell down and sprained | 2 | 76 | 10 | 2 | 3 |
| 4 | Left | Lateral | B | Male | 38 | 4 | Sprained while playing basketball | 4 | 62 | 0.33 | 0.33 | 1 |
| 5 | Left | Lateral | B | Male | 32 | 4 | Sprained while walking | 4 | 68 | 0.75 | 0.75 | 1 |
| 6 | Left | Medial | B | Female | 58 | 4 | Fell down and sprained | 2 | 78 | 10 | 1 | 2 |
| 7 | Left | Lateral | C | Female | 40 | 3 | Injured while deep squating | 3 | 73 | 10 | 2 | 1 |
| 8 | Right | Lateral | C | Male | 32 | 3 | Sprained while playing football | 2 | 76 | 11 | 2 | 2 |
| 9 | Left | Medial | C | Female | 43 | 3 | Sprained while playing football | 3 | 79 | 7 | 3 | 1 |
| 10 | Right | Medial | D | Male | 47 | 3 | Sprained while walking stairs | 2 | 76 | 11 | 1.5 | 2 |
| 11 | Left | Medial | D | Male | 24 | 3 | Sprained while playing  basketball | 3 | 72 | 12 | 2 | 3 |
| 12 | Right | Lateral | D | Female | 46 | 3 | Fell down and sprained | 3 | 70 | 9 | 3 | 1 |
| 13 | Left | Lateral | E | Male | 44 | 4 | Sprained while running | 3 | 73 | 12 | 2.5 | 3 |
| 14 | Right | Lateral | E | Male | 52 | 4 | Sprained while walking stairs | 4 | 63 | 8 | 1 | 2 |
| 15 | Right | Lateral | E | Female | 19 | 4 | Sprained while playing  basketball | 3 | 68 | 9 | 3 | 3 |
